# The Psychological Benefits of Forest Bathing in Individuals with Fibromyalgia and Chronic Fatigue Syndrome/Myalgic Encephalomyelitis: A Pilot Study

**DOI:** 10.3390/healthcare13141654

**Published:** 2025-07-09

**Authors:** Mayte Serrat, Estíbaliz Royuela-Colomer, Sandra Alonso-Marsol, Sònia Ferrés, Ruben Nieto, Albert Feliu-Soler, Anna Muro

**Affiliations:** 1 Unitat d’Expertesa en Síndromes de Sensibilització Central, Hospital de la Vall d’Hebron, 08035 Barcelona, Spain; mayte.serrat@vallhebron.cat; 2 Escoles Universitàries Gimbernat, Universitat Autònoma de Barcelona, 08193 Cerdanyola del Vallès, Spain; sferrespuigdevall@yahoo.es; 3 Department of Clinical and Health Psychology, Universitat Autònoma de Barcelona, 08193 Cerdanyola del Vallès, Spain; estibaliz.royuela@uab.cat (E.R.-C.); albert.feliu@uab.cat (A.F.-S.); 4 IMIM-Institut Hospital del Mar d’Investigacions Mèdiques, 08003 Barcelona, Spain; sandraalon.94@gmail.com; 5 Department of Basic, Developmental and Educational Psychology, Universitat Autònoma de Barcelona, Bellaterra, 08193 Barcelona, Spain; 6 Faculty of Psychology and Educational Sciences, Universitat Oberta de Catalunya, 08035 Barcelona, Spain; rnietol@uoc.edu

**Keywords:** fibromyalgia, chronic fatigue syndrome, forest bathing, shinrin-yoku, forest therapy, mindfulness

## Abstract

Background/Objectives: The main objective of the present study is to assess the short-term effects of Forest Bathing (FB) conducted in a Mediterranean forest on individuals with fibromyalgia (FM) and/or chronic fatigue syndrome/myalgia encephalomyelitis (CFS/ME) on perceived pain, fatigue, state anxiety, positive and negative affect, mood states, and state mindfulness. Methods: A total of 44 participants with FM and/or CSF/ME agreed to participate in this study. The FB session consisted of a 3 km silent walk, lasting three hours and guided by a specialized psychologist and a mountain guide to guarantee the safety of the activity. Paired-sample *t*-tests were used to analyze the pre–post changes in perceived pain, fatigue, state anxiety, positive and negative affect, mood states, and mindfulness. Results: All reported variables but self-reported pain showed statistically significant pre–post variations after the FB session. Particularly, large-to-very-large improvements in positive and negative affect, state anxiety, tension, depression, anger, and vigor were found. Small-to-moderate effect sizes for fatigue, friendliness, and state mindfulness were also reported. Conclusions: This study provides preliminary evidence of the short-term benefits of FB in individuals with FM and/or CFS/ME, especially on state anxiety and negative affect.

## 1. Introduction

In recent years, the role of forests in promoting and improving human health and well-being has been gaining scientific interest [1,2,3]. In this regard, there are many studies supporting the beneficial effects of nature exposure in a broad range of health outcomes and populations, with evidenced positive effects on mental and general health, life satisfaction, longevity, emotional regulation, and cognitive functioning [2,4,5,6]. It has also been reported that the effects of a forest environment induce physiological relaxation, with changes in stress hormones (i.e., cortisol) and the autonomic nervous system [7,8,9,10,11]. The benefits of nature exposure have also been demonstrated for even brief contemplative outdoor activities, such as forest walks and outdoor mindfulness practices, showing a positive effect on mental health in reducing, for instance, depressive and anxiety symptoms [6,12,13].

Different theories exist regarding the association between nature and human health and well-being. For example, the Attention Restoration Theory proposes that spending time in nature can restore attention and prevent the mental fatigue associated with modern lifestyles [14]. Similarly, the Stress Reduction Theory hypothesizes that the connection with nature activates the parasympathetic nervous system and reduces autonomic arousal [15], which is related to stress. In the same vein, the Biophilia hypothesis points to humans naturally desiring to interact with nature and all living things that belong to the natural world [16]. Finally, other explanations suggest that the benefits of nature rely on aspects that individuals encounter when they spend more time in nature, namely, an increase in levels of physical activity, a reduction in exposure to harmful environments (e.g., air pollution, noise, and artificial light), and an increase in exposure to sunlight and vitamin D [17,18].

Among the different nature activities, there is the ancient Japanese practice of shinrin-yoku, also known as Forest Bathing (FB). FB may have remarkable psychological benefits associated with nature immersion, which could be useful for managing and reducing the reported modern “stress state” [5,19]. The FB practice consists of mindful walking in a natural setting, quietly aimed at promoting communication and contact with nature, and it is usually carried out by specialized guides [5]. FB is a form of “Nature Therapy”, defined by Song, Ikei, and Miyazaki [20] as “a set of practices aimed at achieving ‘preventive medical effects’ through exposure to natural stimuli that render a state of physiological relaxation and boost the weakened immune functions to prevent diseases”. Unlike general nature exposure, FB is a structured and mindful practice that involves slow walking, sensory awareness, and guided interaction with the natural environment.

Evidence suggests that FB has psychological and overall health benefits. A recent review indicates that FB may increase human natural killer cell activity; reduce blood pressure and heart rate; diminish stress hormones; balance the autonomic nervous system; improve sleep; reduce anxiety, depression, anger, fatigue, and confusion; and increase vigor [21]. However, the majority of the studies in FB are performed in Asian countries, with small sample sizes and over-representation of young and healthy men; hence, more evidence is needed to confirm the therapeutic effects of forests on health in other biomes around the world and, particularly, in more heterogeneous and clinical populations [5,9,11,13], such as patients with fibromyalgia (SM) syndrome. This allowed us to explore whether the psychological benefits described in Asian settings are also observable in Mediterranean environments, considering both ecological and cultural differences.

FM is a highly prevalent rheumatic syndrome (2% to 8% of the general population) that is usually diagnosed between 20 and 50 years old [22]. It is characterized by chronic, diffuse musculoskeletal pain, fatigue, sleep disorders, high levels of distress, and comorbidity with anxiety and depressive disorders [23]. FM has recently been considered a functional somatic disorder, evolving from a primary pain disorder with a neurobiological basis to a broader biopsychosocial disorder [24]. Although there is no curative treatment for this disease, pain education, cognitive behavioral therapy, and therapeutic exercise are considered adequate approaches to manage FM symptoms [25].

Around 50% of the cases of FM are usually comorbid with other central sensitization syndromes, such as chronic fatigue syndrome/myalgic encephalomyelitis (CFS/ME) [26,27,28,29]. CFS/ME is a disabling and complex illness that affects approximately 1% of the population [30] and is characterized by persistent post-exertional fatigue and substantial symptoms related to cognitive, immune, and autonomic dysfunction, including sore throat, muscle pain, joint pain, weakness, sleep disturbance, memory loss, and poor concentration, impacting individuals’ quality of life and overall well-being [31,32].

FB seems to be a promising intervention for promoting better mental health and wellbeing; however, there is a scarcity of studies exploring its effects in clinical samples. In particular, as far as we know, there is only one study using FB. In this pilot study conducted in Spain by López-Pousa et al. [33], 30 individuals with FM were included and randomly allocated to a group for an aerobic exercise-based program (i.e., 1.25-km walks during six days in a timeframe of 2 weeks) in a mature (*n* = 15) or in a young forest (*n* = 15). Although both groups showed decreases in FM symptoms, the group that performed the walk in the mature forest showed greater improvements in terms of reductions in days with intense pain and insomnia and more days of well-being. Regarding the effects of nature exposure in individuals with CFS/ME, in a recent study including 24 participants with CFS/ME, who were randomly allocated to outdoor daily living activities (for 6 days, at least 3 h each) in a waterfall forest environment or to activities in an urban context, noticed that this natural context compared to urban areas improved their psychological state and enhanced immunity [34].

Accordingly, the main objective of the present study is to assess changes in self-reported pain, fatigue, mental health, and psychological well-being indicators after a 3-h FB session conducted in a Mediterranean forest in a sample of individuals diagnosed with FM and/or CFS/ME. To date, few studies have explored forest bathing in Mediterranean ecosystems or its effects in populations with FM and/or CFS/ME, underscoring the need for pilot data in this specific clinical and environmental context. Given the exploratory nature of this pilot, a single-session format was selected to evaluate the short-term feasibility and immediate psychological effects of FB in this population. We hypothesized that compared to baseline levels, after the FB session, participants would report lower levels of anxiety, negative affect, anger, fatigue, tension, depressive mood, perceived pain, and perceived fatigue, as well as higher levels of positive affect, vigor, friendship, and state mindfulness. Although the “friendliness” dimension was not a primary outcome of interest, it was included as part of the standard POMS scoring and reported for completeness. These mechanisms, including reduction in perceived stress, activation of the parasympathetic nervous system, and enhancement in affective self-regulation, are particularly relevant given the autonomic dysregulation, heightened stress reactivity, and emotional burden often observed in FM and CFS/ME.

## 2. Materials and Methods

### 2.1. Design

A pre–post study design was used, with self-report assessment at baseline and after a 3-h FB session.

### 2.2. Procedure

All participants were recruited by means of social media posting (mainly through an inclusive hiking club association called the CIM project: *Associació Esportiva Projecte Inclusiu CIM*). The activity was provided for free to all individuals with FM and CFS/ME, and participation of their families was also encouraged. The FB session was carried out in the area surrounding Barcelona, on the 10th of October 2021, at 10 a.m. The route started at 41°25′12.3”N 2°05′49.2”E. This area corresponds to the “Baixador de Vallvidrera’’ train station, which is very well-connected with Barcelona center (approx. 15 min by train). “Baixador de Vallvidrera ‘’ is part of the Serra de Collserola, a mountain range of more than 8000 hectares, which is part of the Catalan Coastal Range and located between the rivers Besòs and Llobregat. The vegetation in Collserola is predominantly Mediterranean, consisting of a mix of evergreen and deciduous tree species. It contains many Mediterranean natural environments, predominantly forests, although low vegetation formations (maquis) also abound. In the park, over a thousand major plants and around thirty plant communities have been cataloged, including forests of Aleppo pines and nut pines, evergreen oaklands, riverside copses, maquis and scrublands, brush, and savannah grasslands. The original forest is a coastal holm oak grove (holm oak tree layer with a second shrub layer of common laurustine); however, due to human action (e.g., fires, grassland), most of the mountain range is also covered by Aleppo pines. The forest also features dense undergrowth with a variety of herbaceous plants, including wildflowers, grasses, and ferns. In addition to its natural features, the Collserola forest also serves as a recreational area for outdoor activities such as hiking, mountain biking, and nature observation.

A masked unipersonal code was given to each participant, and then a link to an online questionnaire was sent by email. Participants answered the baseline questionnaire approximately 1 h before starting the FB and in the first 24 h after the FB session.

### 2.3. Description of the FB Session

The session was guided by one specialized psychologist with expertise in FB (A.M.), a psychologist and physiotherapist specializing in fibromyalgia (M.S.), and three mountain guides to guarantee the safety of the activity. The FB activity consisted of a 3 km silence walk in 3 h, with the realization of three 10 min stops (i.e., before starting the walk, in the middle, and at the end of it) with practices of deep breathing and mindful awareness of the five senses in nature. A similar FB protocol was used in the study by Muro et al. [13].

### 2.4. Sample

A total of 44 participants with FM and/or CSF/ME agreed to participate in this study. The inclusion/exclusion criteria were to be diagnosed with FM, SFC/ME, or both syndromes by a rheumatologist; to be more than 18 years of age; to be able to understand Spanish; and to be able to provide written informed consent.

All participants consented to collaborate voluntarily and provided informed consent before participating in the study. The ethics committee of the Autonomous University of Barcelona approved the study in advance (reference code UAB5339).

### 2.5. Instruments

The survey was designed to gather data about the following:

Sociodemographic and clinical characteristics: age, body mass index (BMI), educational level, site of residence (urban or rural), illness self-perceived start (ISPS), and incapacity certificate. Furthermore, physical exercise habits (i.e., weekly amount of practice, exercise intensity, and type of physical exercise) were also recorded.

The Depression Anxiety Stress Scale—21 (DASS-21) [35] was administered to assess depressive, anxiety, and stress symptoms of the sample at baseline. The DASS-21 is a 21-item self-reported scale that assesses symptoms of depression, anxiety, and stress (7 items each). Each item is scored from 0 (did not apply to me at all over the last week) to 3 (applied to me very much or most of the time over the past week) and asks for symptoms presented over the previous week. Total scores on each subscale may range from 0 to 21, with higher scores indicating a more severe status. The Spanish adaptation of the DASS-21 has an adequate internal consistency (with Cronbach’s alphas of 0.84, 0.70, and 0.82 for depression, anxiety, and stress, respectively) [36].

The 20-item subscale for assessing state anxiety levels (“right now”) from the State–Trait Anxiety Inventory—State (STAI) [37,38] was used. It is a 4-point Likert scale ranging from 1 (not at all) to 4 (very much so). High scores correspond to altered states related to anxiety, and low scores suggest an absence of stress and emotional stability. The Spanish version of the STAI state showed an internal consistency of Cronbach’s α = 0.94 [37].

The Positive Affect and Negative Affect Scale (PANAS) [39] was used. It consists of two subscales of 10 items each for assessing positive and negative affect using a 5-point scale from 1 (very slightly or not at all) to 5 (extremely). Scores of each subscale can range from 10 to 50, with higher scores meaning higher positive and negative affect levels. The scale has shown good psychometric properties in Spanish samples [40], with internal consistency indexes (Cronbach’s alpha) of 0.92 for the Positive Affect Scale and 0.88 for the Negative Affect Scale.

The State Mindfulness Scale (SMS) [41] consists of 21 items, with a 5-point response scale that ranges from 1 (not at all) to 5 (very well) to evaluate the degree of conformity, with the proposed assertions related to their experiences in the previous 15 min. It is focused on two dimensions: mindfulness state of mind (related to thoughts) and mindfulness state of body (related to physical sensations). Higher scores represent a higher level of state mindfulness. In this study sample, this test showed an optimal internal consistency of Cronbach’s α = 0.97, consistent with data obtained by Tanay and Bernstein, who reported strong internal consistency reliabilities (α = 90–95).

The Profile of Mood States (POMS) [42,43] consists of 30 items measuring six mood states (anger, fatigue, vigor, friendship, tension, and depressive). Participants are requested to rate each item using a 5-point Likert scale from 0 (*not at all*) to 4 (*extremely*). Higher scores indicate higher levels of that mood state. POMS subscales of the Spanish validation showed a Cronbach’s α of between 0.78 (friendship) and 0.87 (fatigue).

A Visual Analog Scale for rating pain intensity in the present moment (VAS PAIN) [44], with extremes from 0 (no pain) to 10 (unbearable pain), was used. Similarly, a VAS for assessing fatigue (VAS FATIGUE) [44], with extremes from 0 (i.e., lots of energy) to 10 (i.e., no energy), was used.

### 2.6. Statistical Analyses

Descriptive statistics such as means (with standard deviations) and frequencies were calculated for the sociodemographic and clinical characteristics. Paired-sample *t*-tests were used to analyze the pre–post changes in outcome measures before and after the FB. For a more precise interpretation of the relevance of the results in each assessed domain, effect sizes for pre–post changes were also calculated using Cohen’s *d* (rule of thumb for Cohen’s *d*: 0.2 = small, 0.5 = medium, and 0.8 = large effect sizes). All the statistical analyses were conducted using the IBM SPSS Statistics for Windows, version 21 (IBM Corp., Armonk, NY, USA) program, assuming a statistical significance level of 5%.

## 3. Results

Most of the participants had been diagnosed with both FM and CFS/ME (*n* = 37, 80.4%). Three participants had only an FM diagnosis (6.5%) and five only a CFS/ME diagnosis (10.9%). All participants were women, with a mean age of 54 years, ranging from 26 to 69, with a BMI of 28.31 ± 6.46. Most of them (93.2%) lived in urban areas and had achieved secondary or higher education (61.4%). They had an average of 11–13 years of self-perceived illness start/duration and showed a moderate-to-high levels of pain (scoring 6–7 out of 10) and fatigue (scoring 7–8 out of 10). More than half (56.8%) had a recognized disability degree of 33% or more. According to the DASS-21 standard cut-offs [35], 47.7% of the sample rated severe or extremely severe for Depression, 53.5% for Anxiety, and 31.8% for Stress. In addition, participants reported that they were not very physically active individuals, as 84.1% of the participants performed light exercise (such as walking) twice a week (see Table 1).

Most of the psychological indicators showed statistically significant pre–post variations after the intervention (see Table 2). More precisely, there was a statistically significant decrease in negative affect (*p* < 0.001; *d* = 0.84), state anxiety (*p* < 0.001; *d* = 1.31), anger (*p* < 0.001; *d* = 0.84), fatigue (*p* = 0.009; *d* = 0.40), tension (*p* < 0.001; *d* = 1.05), and depression (*p* < 0.001; *d* = 1.21). Moreover, a significant increase in positive affect (*p* < 0.001; *d* = 0.85), vigor (*p* < 0.001; *d* = 0.84), friendship (*p* = 0.001; *d* = 0.44), mindfulness of body (*p* < 0.001; *d* = 0.48), and mindfulness of mind (*p* < 0.001; *d* = 0.64) was noted. There was also a significant decrease in fatigue levels (*p* = 0.003; *d* = 0.46) after the FB. There were no significant differences in pain intensity (*p* = 0.421; *d* = 0.14).

## 4. Discussion

The main aim of this study was to assess whether an FB session could improve several outcomes in individuals with FM and/or SFC, with a particular focus on mental health and well-being. These results are strengthened given the notable clinical severity of the individuals included, with moderate-to-high reported levels of pain and fatigue (cardinal symptoms of FM and CFS/ME), a high rate of recognized disability degree, and high depressive and anxious symptomatology. Furthermore, regarding the generalizability of the findings, more than 80% of the participants reported that they only performed light exercise (such as walking) twice a week, so we can ascertain that not only very physically active (a behavioral pattern not usually seen in these conditions) individuals were enrolled in this study.

Overall, the results obtained in this pilot study align with previous studies in Asian and Mediterranean forests and show how FB may help in fostering mental well-being [2,5,13,19,45]. The current study found a decrease in negative affect, state anxiety, anger, fatigue, tension, and depression, and an increase in positive affect, vigor, friendship, and mindfulness state in a clinical sample of individuals showing FM and/or SFC/ME. However, contrary to expectations, this study did not find a significant improvement in self-reported pain following the FB session. This study’s results are also in line with previous studies that reported increased well-being after contact with nature, especially following an FB session [1,3,18,46]. As these studies suggest, FB might produce benefits because it increases exposure to nature, which is related to a normalization of the stress-response system. In addition, our results also indicated an increase in state mindfulness, consistent with the Attention Restoration Theory [14]. According to this theory, spending time in nature has health benefits since it decreases attentional fatigue caused by the modern lifestyle. Therefore, the mindful state generated by the FB practice might have enabled individuals to enjoy the present moment and focus on the environment, the exercise, or the silent company of other people instead of on their distressing internal states or engaging in ruminating tendencies. Furthermore, our results showed increased friendliness following the FB practice, which has been previously documented in some studies [13,17,47]. In other words, FB may provide a shared experience with a space for socialization in which new bonds and connections may be created, which might increase social support. These findings highlight the potential of FB as a holistic intervention that not only promotes physical and mental health through nature exposure but also fosters mindfulness and social connection, contributing to overall well-being.

It is important to note that most of the studies mentioned above were conducted with healthy populations, often composed of young men and in Asia. Therefore, our results allow us to extend the positive effects of FB to a more specific population: patients with somatic functional syndromes such as FM and CFS/ME from the Mediterranean area. In this sense, our results are consistent with data obtained in the study of Muro et al. [2] in which they concluded that FB in a Mediterranean forest increases psychological well-being in a sample of healthy adults compared to a control hiking group.

To our knowledge, only one study explored the benefits of walking in a Mediterranean forest among patients with FM. Unlike our study, which lasted only one session and was conducted in a relatively young forest (i.e., due to recent human action (e.g., fires, grassland), most of the mountain range is covered by Aleppo pine forests and not by its original coastal holm oak grove forest), the Lopez-Pousa et al. [33] study explored a two-week intervention in two types of Mediterranean forests (mature and young) among FM patients. Lopez-Pousa et al. [33] concluded that an aerobic exercise program consisting of walking through a forest could reduce FM symptoms. But only in the mature forest did participants experience a subjective perception of having fewer days of pain and insomnia and more days of wellness. Unlike them, we did not find differences in terms of pain. This could be because our FM session only lasted 3 h, and perhaps more time is needed to obtain results in this highly resistant clinical outcome. Therefore, the duration of nature exposure could be a major factor influencing the effectiveness of the intervention, as has been previously suggested by Antonelli et al. [46].

Regarding the pain outcome, contrary to our results, Han et al. [48] reported changes in pain perception following forest therapy among a sample of adults who suffered from chronic pain. There are several differences between our study and the study of Han et al. [48] that could explain the discrepancy in the results. The intervention of Han et al. [48] lasted for two days and included other components apart from FB, such as psychoeducation and time for socialization. It can therefore be assumed that the FB intervention might be more beneficial when combined with other types of activities and when it has a longer duration.

Another promising result observed after FB was the significant improvement in the CFS/ME core symptom of fatigue (moderate effect size) and, in parallel, the opposite aspect of vigor (large effect size). This result is in line with other research on the benefits of walking in the woods to reduce fatigue in FM and CFS/ME [33,34]. Forest bathing, by providing a sensory-rich environment that engages multiple modalities of perception within a natural context in the forest, has been shown to stimulate the parasympathetic nervous system and reduce sympathetic activity, resulting in a state of greater well-being and vitality [49].

### Limitations and Future Directions

The present study has some strengths that deserve to be stated. Mainly, the sample size (*n* = 44) was larger than that of the other study conducted on individuals with FM (*n* = 30) [33], and the evaluation protocol included a thorough evaluation of the impact of the FB session at the mental health and well-being level. Furthermore, the intervention was conducted by a multidisciplinary team including two specialized psychologists and a mountain guide. However, the present study presents limitations that should also be acknowledged. The first one is not having a control group, which impedes establishing causality. The inclusion of a control group (e.g., a psychoeducation control group) will be considered in future studies in order to assess the effectiveness of the intervention. Another consideration was the short duration of the FB protocol (only one session) and the lack of a follow-up assessment to evaluate the stability of the observed improvements. Furthermore, further studies should also evaluate potential moderators (e.g., gender, age, physical fitness) and mediators (e.g., self-efficacy, self-esteem, nature connectedness) in explaining the beneficial effects of FB. In addition, the sample included only women. Although our results are interesting for generalizing those of previous studies that only included males, future studies should extend and compare the benefits of FB in a clinical sample of men and women. Additionally, recruitment via social media and a mountaineering club may have introduced a self-selection bias toward individuals already positively inclined to nature-based experiences. Moreover, the absence of a control group and the use of a single FB session limit the robustness of our conclusions and prevent us from evaluating long-term or cumulative effects. In addition, environmental exposure data (e.g., vegetation density, soundscape, air quality) were not recorded, which limits the characterization of the forest setting. Future studies should consider including physiological indicators such as HRV to support the self-reported outcomes.

In conclusion, it seems that FB is an affordable practice that may be useful for managing symptomatology in patients with FM and CFS/ME. In contrast, pain outcomes did not show significant improvement. This may be due to the chronic and multifactorial nature of pain in these conditions, which likely requires repeated or more intensive interventions. Further research is needed to evaluate its effectiveness compared to other existing therapeutic approaches and to delve further into mechanisms of change associated with FB benefits.

## 5. Conclusions

The current study aimed to explore nature’s benefits on individuals with FM and/or CFS/ME. The results from this study indicated that a 3-h session of FB, conducted in a Mediterranean forest, may have a positive short-term impact on some aspects of mental health and psychological well-being for patients with these characteristics. FB could be an accessible and low-cost add-on therapeutic approach that could be integrated into standard treatments for chronic diseases such as FM and CFS/ME. Further randomized controlled trials comparing the effects of FB with other recognized therapeutic approaches for FM and/or CFS/ME (e.g., cognitive behavioral therapy, indoor therapeutic exercise) and including longer follow-ups are needed. Furthermore, moderators (e.g., session duration) and mediators of change (e.g., changes in state mindfulness) of FB should also be explored in future studies.

## Figures and Tables

**Table 1:** Sociodemographic and clinical characteristics of the study sample.

	Total Sample
	(*n* = 44)
Sociodemographics	
Age (years), *M* ± *SD*	53.82 ± 8.57
Women, n (%)	44 (100)
Only fibromyalgia	3 (6.80)
Only CFS/ME	4 (9.10)
Fibromyalgia and CFS/ME diagnosis	36 (81.80)
BMI, *M* ± *SD*	28.31 ± 6.45
Educational level, *n* (%)	
Primary education	13 (29.50)
Secondary education	16 (36.40)
Higher education	11 (25.00)
Other	4 (9.10)
Residence, *n* (%)	
Urban area	41 (93,20)
Rural area	3 (6.80)
Clinical characteristics	
VAS Pain, *M* ± *SD*	6.64 ± 1.88
VAS Fatigue, *M* ± *SD*	7.48 ± 1.42
Fibromyalgia ISPS (years), *M* ± *SD*	13.49 ± 10.89
CFS/ME ISPS (years), *M* ± *SD*	10.93 ± 10.01
Incapacity certificate, *n* (%)	
No	12 (27.30)
Requested	7 (15.90)
Between 33% and 66%	21 (47.70)
More than 66%	4 (9.10)
DASS Depression *, *n* (%)	
Normal 0–9	15 (34.10)
Mild 10–13	4 (9.10)
Moderate 14–20	4 (9.10)
Severe 21–27	7 (15.90)
Extremely severe +28	14 (31.80)
DASS Anxiety *, *n* (%)	
Normal 0–9	12 (27.30)
Mild 10–13	2 (4.50)
Moderate 14–20	6 (13.60)
Severe 21–27	7 (15.90)
Extremely severe +28	17 (38.60)
DASS Stress, *n* (%)	
Normal 0–9	14 (31.80)
Mild 10–13	7 (15.90)
Moderate 14–20	9 (20.50)
Severe 21–27	8 (18.20)
Extremely severe > 28	6 (13.60)
Physical exercise habits	
*Weekly amount of physical exercise, n (%)*	
<1 h per week	10 (22.70)
1–2 h per week	13 (29.50)
2–3 h per week	10 (22.70)
>3 h per week	8 (18.20)
No exercise	3 (6.80)
*Intensity of weekly exercise sessions, n (%)*	
Very light	14 (31.80)
Mild	18 (40.90)
Moderate	8 (18.20)
*Physical exercise type, n (%)*	
Walking	37 (84.10)
Yoga	1 (2.30)
Nordic walking	1 (2.30)
Other	3 (6.80)

Note: * levels according to DASS-21 standard cut-offs (Lovinbond & Lovibond, [35]).

**Table 2:** Observed changes after forest-bathing practice.

	*M* (*SD*) Pre	*M* (*SD*) Post	Pre-Post Diff.	95% CI	*t*	*p*	*d*
	(*n* = 44)	(*n* = 42)	*M* (*SD*)				
VAS	6.64 (1.88)	6.31 (2.62)	0.33 (2.63)	−0.48, 1.13	0.813	0.421	0.14
Pain							
VAS Fatigue	7.48 (1.42)	6.36 (2.76)	1.12 (2.31)	0.41, 1.83	3.167	0.003	0.46
PANAS Positive Affect	25.52 (8.91)	34.07 (9.56)	−7.98 (6.72)	−10.05, −5.91	−7.779	<0.001	0.85
PANAS Negative Affect	22.84 (10.00)	14.80 (6.73)	7.49 (8.60)	4.84, 10.13	5.713	<0.001	0.84
STAI Anxiety state	31.59 (14.54)	13.47 (11.71)	17.63 (12.62)	13.74, 21.51	9.162	<0.001	1.31
POMS Anger	5.11 (5.40)	1.23 (2.78)	4.09 (5.34)	2.45, 5.74	5.022	<0.001	0.84
POMS Fatigue	12.18 (5.21)	9.85 (6.07)	2.35 (5.62)	0.62, 4.08	2.739	<0.01	0.4
POMS Vigour	6.36 (4.57)	10.69 (4.97)	−4.09 (4.33)	−5.43, −2.76	−6.198	<0.001	0.84
POMS Friendliness	14.54 (4.52)	16.69 (3.78)	−1.93 (3.55)	−3.02, −0.84	−3.567	0.001	0.44
POMS Tension	9.29 (6.70)	2.71 (4.00)	6.35 (5.33)	4.71, 7.99	7.816	<0.001	1.05
POMS Depression	9.63 (6.30)	2.80 (3.76)	6.53 (6.34)	4.58, 8.49	6.762	<0.001	1.21
SMS Mindf. body	23.22 (5.38)	25.80 (4.37)	−2.40 (4.11)	−3.66, −1.13	−3.820	<0.001	0.48
SMS Mindf. mind	55.13 (13.09)	63.42 (11.35)	−8.02 (9.12)	−10.83, −5.22	−5.766	<0.001	0.64

## Data Availability

The raw data supporting the conclusions of this article will be made available by the authors on request.
